# Characterization of *Toxoplasma gondii* glyoxalase 1 and evaluation of inhibitory effects of curcumin on the enzyme and parasite cultures

**DOI:** 10.1186/s13071-015-1268-5

**Published:** 2015-12-23

**Authors:** Youn-Kyoung Goo, Junya Yamagishi, Akio Ueno, Mohamad Alaa Terkawi, Gabriel Oluga Aboge, Dongmi Kwak, Yeonchul Hong, Dong-Il Chung, Makoto Igarashi, Yoshifumi Nishikawa, Xuenan Xuan

**Affiliations:** Department of Parasitology and Tropical Medicine, Kyungpook National University School of Medicine, Daegu, 700-422 Republic of Korea; Research Center for Zoonosis Control, Hokkaido University, Sapporo, Hokkaido 001-0020 Japan; National Research Center for Protozoan Diseases, Obihiro University of Agriculture and Veterinary Medicine, Obihiro, Hokkaido 080-8555 Japan; Department of Public Health, Pharmacology and Toxicology, University of Nairobi, P.O. BOX 29053, 00625 Kangemi, Nairobi Kenya; Department of Veterinary Medicine, College of Veterinary Medicine and Stem Cell Research Therapeutic Institute, Kyungpook National University, Daegu, 700-701 Republic of Korea

**Keywords:** *Toxoplasma gondii*, Glyoxalase pathway, Curcumin

## Abstract

**Background:**

The glyoxalase pathway, which includes two enzymes, glyoxalase 1 and 2 (Glo1 and Glo2), is a ubiquitous cellular system responsible for the removal of cytotoxic methylglyoxal produced during glycolysis. Protozoan parasites, including *Toxoplasma gondii* (*T. gondii*) tachyzoites, produce methylglyoxal because of increased glycolytic fluxes. A Glo1 inhibitor such as curcumin could be considered a drug candidate for anti-protozoan, anti-inflammatory, and anti-cancer therapy.

**Methods:**

The *T. gondii* Glo1 gene (TgGlo1) was cloned and the recombinant protein was produced. Enzyme kinetics of TgGlo1 and five mutants were evaluated by adding methylglyoxal and glutathione to a reaction mixture. Finally, the inhibitory effects of various concentrations of curcumin on recombinant TgGlo1 were evaluated using in vitro cultures of *T. gondii*.

**Results:**

Active recombinant TgGlo1 was successfully produced and the active sites (E166 and E251) of TgGlo1 were verified by point mutagenesis. Curcumin at the tested doses inhibited the enzymatic activity of recombinant TgGlo1 as well as the parasitic propagation of in vitro-cultured *T. gondii*. The K_i_ and IC_50_ were 12.9 ± 0.5 μM and 38.3 ± 0.9 μM, respectively.

**Conclusion:**

The inhibitory effect of curcumin on the enzymatic activity of TgGlo1 and parasitic propagation of *T. gondii* could be explored in the potential development of a potent drug for the treatment of toxoplasmosis. However, considering the fact that curcumin is known to have many effects on other molecules in the micromolar range, further elucidation of curcumin’s direct inhibition of the glyoxalase system of *T. gondii* will be needed.

**Electronic supplementary material:**

The online version of this article (doi:10.1186/s13071-015-1268-5) contains supplementary material, which is available to authorized users.

## Background

*Toxoplasma gondii* is a major pathogen of a broad range of warm-blooded animals, including humans, livestock, and domestic pets [1, 2]. The parasite is of clinical importance in causing devastating disease in the developing fetus, as well as opportunistic infections in immunocompromised and transplant patients [3]. In human hosts, the parasites differentiate into tachyzoites and bradyzoites, which display distinct physiological features. The dormant bradyzoites show an extremely reduced growth rate, close to complete arrest of the cell cycle, while tachyzoites rapidly duplicate in the acute phase of infection. The tachyzoite stage constitutes an effective pathway for nutrient acquisition and energy metabolism for the parasite.

The glyoxalase pathway is the ubiquitous cellular system responsible for the removal of methylglyoxal, a cytotoxic reactive carbonyl compound produced in many organisms as a consequence of central metabolism [4–6]. During glycolysis, methylglyoxal is produced by imperfect degradation of glucose because of the spontaneous elimination of phosphate from dihydroxyacetone-phosphate or glyceraldehyde-3-phosphate [7]. *Plasmodium* species, which are apicomplexan parasites closely related to *T. gondii*, also show increased methylglyoxal production [8, 9]. Therefore, the increased glycolytic fluxes in *T. gondii* tachyzoites would suggest that these parasites require an efficient detoxification system for removal of harmful methylglyoxal. While there are several reports of studies focusing on the glyoxalase system of *Plasmodium* species as a potential drug target, no such study has been reported in *T. gondii* to date [10].

Curcumin is a polyphenol derivative isolated from the plant *Curcuma longa*. By interacting with more than 30 proteins, curcumin exhibits anti-inflammatory, anti-oxidant, anti-viral, and anti-infectious activity in extensive studies conducted over the past several decades [11]. Curcumin was recently reported to be a non-glutathione inhibitor of small homodimeric mammalian glyoxalase 1 (Glo1) [12]. In addition, curcumin showed in vitro and in vivo anti-trypanosomal effects in *Trypanosoma evansi* as well as anti-malarial effects in *Plasmodium falciparum* cultures and in *Plasmodium berghei* in vivo [13–15]. In these studies, the effects of curcumin appeared to target the glyoxalase system, as suggested by its inhibition of the enzymatic activities of Glo1 of the *Trypanosoma* and *Plasmodium* species.

In the present study, we identified a functional Glo1 of *T. gondii* (TgGlo1) and evaluated active sites of TgGlo1 by point mutagenesis and enzyme kinetic analysis. In addition, inhibitory effects of curcumin against the enzymatic activity of recombinant TgGlo1 and parasite growth in *T. gondii* cultures were analyzed.

## Methods

### Parasites and cell cultures

The *T. gondii* RH and green fluorescent protein-expressing RH (GFP-RH) strains used in this study were maintained in Vero cells and human foreskin fibroblasts (HFFs) at 37 °C and 5 % CO_2_ in Dulbecco’s modified eagle medium (DMEM, Sigma-Aldrich, St. Louis, MO USA) supplemented with 10 % heat-inactivated fetal bovine serum (FBS).

### Cloning of the TgGlo1 gene

TgGlo1 was amplified from *T. gondii* cDNA using the primers listed in Table 1. The specific primers were designed according to the TgGlo1 sequence in ToxoDB (accession number, TGGT1_248400). The amplified polymerase chain reaction (PCR) products were extracted using the QIAquick gel extraction kit (QIAGEN, Hilden, Germany) and then digested with *Eco*RI and *Hin*dIII. The digested DNA was inserted between the *Eco*RI and *Hin*dIII sites of the expression vector pRSETb (Invitrogen, Carlsbad, CA, USA). Thereafter, accurate gene insertion was confirmed by digestion with restriction enzymes and sequencing.

**Table 1:** Primers used for amplification of TgGlo1 gene and generation of point mutations of TgGlo1

Gene	Primer	Sequence
TgGlo1	Forward	5′-GGGGAATTCATGTCCAGAAGTTTGCCC-3′
	Reverse	5′-GGGAAGCTTTTACTTCTTTGAAAGGAACG-3′
E92Q	Forward	5′-GGGACGTGCTTGCAACTCACTCACAATC-3′
	Reverse	5′-GATTGTGAGTGAGTTGCAAGCACGTCCC-3′
E166Q	Forward	5′-GGATACTGGATACAATTGGTTTCTCGG-3′
	Reverse	5′-CCGAGAAACCAATTGTATCCAGTATCC-3′
R188E	Forward	5′-CAGACGATGATTGAAATCAAAGATCCG-3′
	Reverse	5′-CGGATCTTTGATCCGAATCATCGTCTG-3′
E251Q	Forward	5′-CAGCCTGTATTACAACTCACACACAATCAC-3′
	Reverse	5′-GTGATTGTGTGTGAGTTGTAATACAGGCTG-3′
E324Q	Forward	5′-GGATATAGCATCCAACTGATTCAG-3′
	Reverse	5′-CTGAATCAGTTGGATGCTATATCC-3′

### Generation of TgGlo1 point mutants

Mutations of the TgGlo1 gene (E92Q, E166Q, R188E, E251Q, E324Q, E92Q/E324Q, and E166Q/E251Q) were introduced by PCR using Ex Taq polymerase (Takara, Shiga, Japan) with mutated primers (Table 1) and the TgGlo1 gene as a template. The mutant genes were subcloned into the pGEM T-easy vector (Promega, Madison, WI, USA) and subsequently confirmed by sequencing both strands. The mutated genes were subcloned into the pRSETb vector (Invitrogen) for recombinant protein expression.

### Expression and protein purification of wild-type and mutants of TgGlo1

The protein expression was performed as previously described by Deponte et al*.* [16] with modifications. The pRSETb constructs of the wild-type and mutant TgGlo1 genes were expressed in *E. coli* strain BL21(DE3)pLysS (Invitrogen). Transformation of the respective plasmid to competent cells was performed before each expression, and the bacteria were precultured overnight at 37 °C. The preculture was added to fresh medium (10 g/L yeast extract, 5 g/L peptone, 10 g/L NaCl, 2 g/L MgSO_4_ · 7H_2_O, 10 mM 3-(*N*-morpholino) propanesulfonic acid (MOPS)/NaOH at pH 7.5, and 100 mg/L ampicillin) and grown at 37 °C. After induction by 0.1 mM isopropyl β-d-1-thiogalactopyranoside and overnight culturing, 3 l of culture cells were harvested by centrifugation (10 min, 12,000 × *g*) and resuspended in 20 mL of buffer containing 10 mM MOPS/NaOH (pH 7.8), lysozyme, DNase I, and 50 μM phenylmethanesulfonyl fluoride. Then, the resuspended cells were sonicated at 4 °C and centrifuged for 30 min at 12,000 × *g*. The supernatant was applied to an *S*-hexylglutathione-agarose column (Sigma-Aldrich), which was equilibrated with buffer containing 10 mM MOPS/NaOH (pH 7.8). After washing with eight column volumes of 10 mM MOPS/NaOH and 200 mM NaCl (pH 7.8), the recombinant enzyme was eluted by three column volumes of 5 mM *S*-hexylglutathione (pH 7.8). The eluent was loaded onto a nickel-nitrilotriacetic acid column (Qiagen) and eluted with buffer containing 125 mM imidazole (pH 8.0). The purity of rTgGlo1 was confirmed by SDS-PAGE (Additional file 1: Figure S1).

### Production of antiserum against TgGlo1

The antiserum specific to TgGlo1 was produced by immunizing five 6-week-old female ICR mice (CLEA Japan, Inc., Tokyo, Japan). The purified rTgGlo1 (200 μg) were emulsified with an equal volume of complete Freund’s adjuvant (Difco Laboratories, Detroit, MI, USA), and then intraperitoneally injected. Two additional boosters with 100 μg of each antigen with incomplete Freund’s adjuvant (Difco) were intraperitoneally administered at 2-week intervals. The mice were bled 2 weeks after the last booster, and serum samples were stored at −30 °C. The specificity of anti-rTgGlo1 serum was evaluated by western blot as shown in Additional file 1: Figure S2.

### Steady-state kinetics

The steady-state kinetics of the recombinant enzymes were monitored spectrophotometrically, using a thermostatted Hitach U-2001 visible spectrophotometer. Experiments to determine K_m_ values were performed as described previously [16]. Stock solutions of 10 mM glutathione (GSH; Sigma-Aldrich) in assay buffer containing 50 mM MOPS/NaOH (pH 7.0) were freshly prepared before each experiment. For the desired concentration of hemithioacetal, concentrations of methylglyoxal and GSH in 1 mL of the assay mixture were calculated using the following equation.

$$ \mathrm{K}\mathrm{d} = 3\ \mathrm{m}\mathrm{M} = \left(\left[\mathrm{methylglyoxal}\right]\left[\mathrm{G}\mathrm{S}\mathrm{H}\right]\right)/\left[\mathrm{hemithioacetal}\right] $$

The calculated concentrations of free GSH after 5 min of preincubation and hemithioacetal were 0.1 mM and 5–500 μM, respectively. After the addition of enzyme, the formation of *S*-d-lactoylglutathione was measured spectrophotometrically at 240 nm (ε = 3.37 mM^−1^cm^−1^). The final assay concentration of the wild-type enzyme and mutants was 5–200 nM. The kinetic data of the initial reaction velocities were plotted according to the Lineweaver-Burk equation using the program SigmaPlot 10.0 (Systat Software Inc., San Jose, CA, USA).

### Enzymatic activity inhibition assays of curcumin

The curcumin used for this assay was purchased from Sigma-Aldrich. Enzymatic activity of recombinant TgGlo1 was monitored at 30 °C using a thermostatted spectrophotometer. The TgGlo1 activity was determined at 240 nm by measuring the conversion of the hemithioacetal (the product of methylglyoxal and GSH) into *S*-d-lactoylglutathione (ε = 3.37 mM^−1^cm^−1^). Stock solutions of 10 mM curcumin in assay buffer (50 mM MOPS/NaOH pH 7.0) and less than 10 mM curcumin in methanol were freshly prepared before each experiment. The curcumin was added after the first 4 min of the 5-min preincubation period and the reaction was initiated by adding 5 nM of enzyme.

### Growth inhibition assay of curcumin-treated *T. gondii*

Growth inhibition assay was performed following previously described methods with modifications [17–19]. Inhibition of parasite growth by curcumin was determined by counting the number of vacuoles containing 1, 2, 4, 8, or more than 16 tachyzoites. To investigate the effects of curcumin exposure on parasites, HFFs in 96-well plates were infected for 4 h and then treated with vehicle or decreasing concentrations (25, 15, 10, 5, 2.5, 1, and 0.1 μM) of curcumin for 48 h. Plates were harvested and analyzed by counting the number of vacuoles with different dividing stages of tachyzoites. In addition, in order to determine the 50 % inhibitory concentration (IC_50_) of curcumin against *T. gondii* cultures, HFFs infected with *T. gondii* expressing GFP were counted under a microscope. The IC_50_ represents the concentration of curcumin that was required for inhibition of 50 % of *T. gondii* proliferation in the HFF.

### Ethics statement

This study and all procedures were approved by the Animal Care and Use Committee of Obihiro University of Agriculture and Veterinary Medicine, Japan. The experimental animals were housed, fed, and given clean drinking water in accordance with the stipulated rules for the Care and Use of Research Animals Promulgated by Obihiro University of Agriculture and Veterinary Medicine, Japan.

## Results and discussion

### Characterization of *T. gondii* glyoxalase 1

**Fig. 1:**
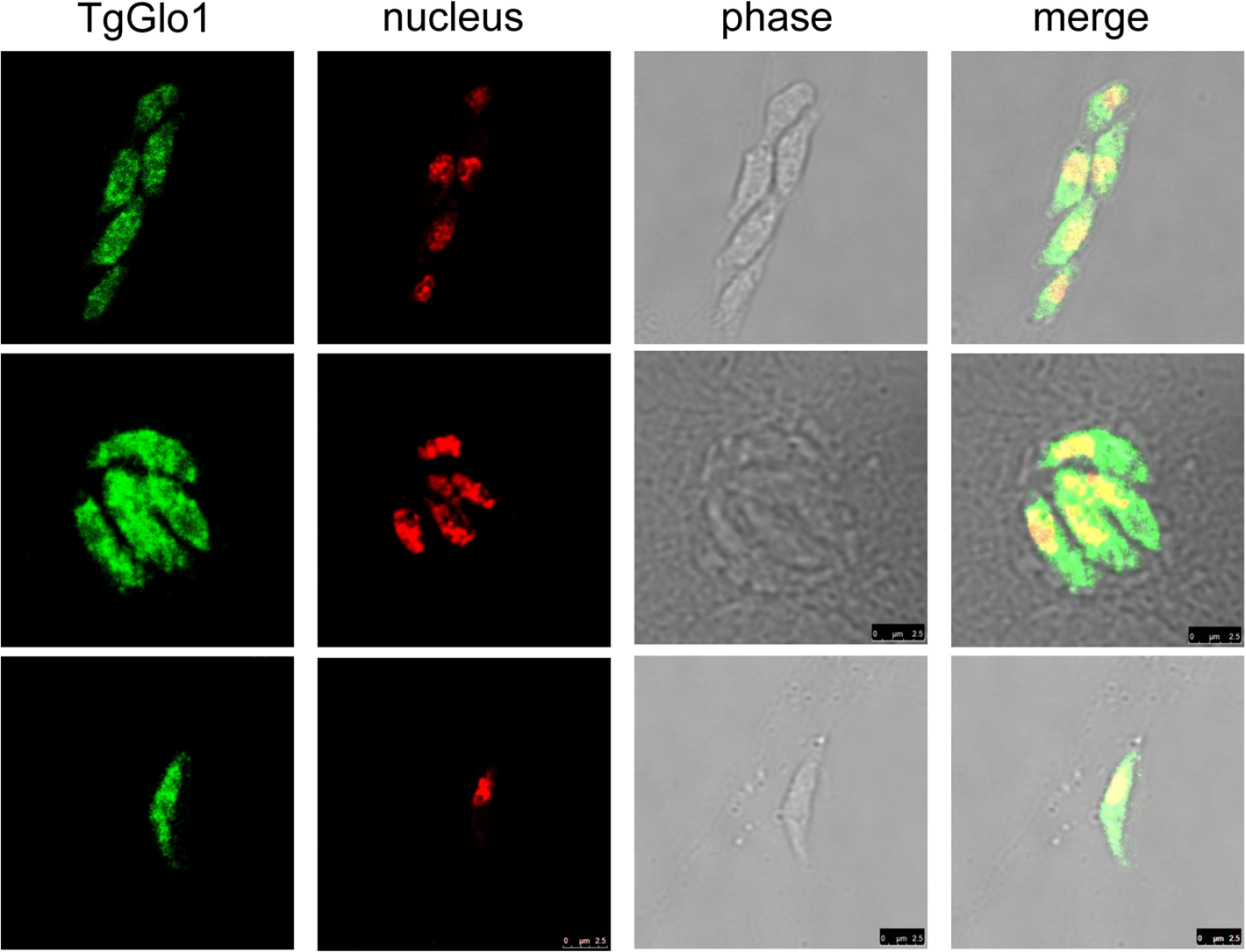
Immunofluorescence microscopy analysis. The specific reaction of the rTgGlo1 and mouse anti-rTgGlo1 serum is green and the nuclei are red. Merged image of fluorescent green reactivity and red PI staining of nuclei with phase-contrast images of the parasites

TgGlo1 was amplified from cDNA of a *T. gondii* RH strain and contained a single open reading frame of 1008 nucleotides encoding a polypeptide of 336 amino acid residues. The sequence of TgGlo1 was identical to that previously reported (GenBank accession no. EPR63516). The purity of rTgGlo1 was confirmed by SDS-PAGE (Additional file 1: Figure S1). Two putative glyoxalase domains were identified at 20–167 and 181–325 amino acids, by an open-sourced gene analysis program, SMART (http://smart.embl-heidelberg.de/). TgGlo1 shared homology with Glo1 of *Neospora caninum* (90 %), *Eimeria acervulina* (64 %), *Eimeria tenella* (61 %), and *P. falciparum* (50 %). No signal peptide was predicted for TgGlo1 by SMART, indicating that it is likely a cytosolic protein. This notion was further supported by the immunofluorescence assay with antiserum against TgGlo1 using confocal microscopy, which revealed that TgGlo1 was located in the cytoplasm of *T. gondii* (Fig. 1), while pre-immune serum did not react with *T. gondii* parasites (Additional file 1: Figure S3).

### Evaluation of enzyme active sites of *T. gondii* glyoxalase 1

**Fig. 2:**
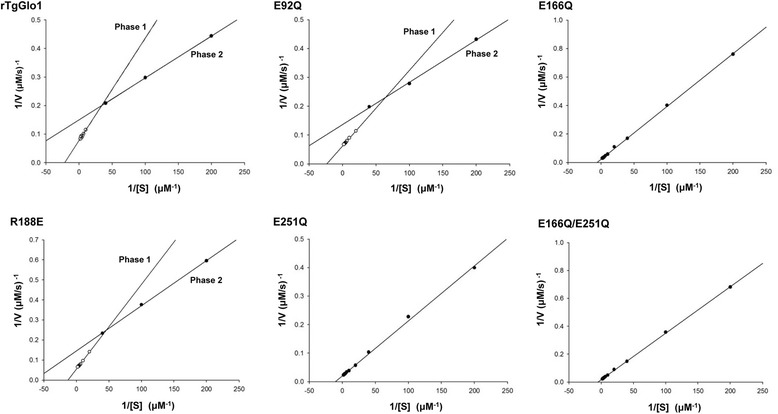
Steady-state kinetics of wild-type rTgGlo1 and five mutants, E92Q, E166Q, R188E, E251Q, and E166Q/E251Q. Measurements are expressed as the mean of three independent experiments. The values for wild-type rTgGlo1, E92Q, and R188E were obtained from two phases (phase 1, 1/[S] = less than 40 μM; phase 2, 1/[S] = greater than 40 μM)

In order to evaluate the enzymatic activity of recombinant TgGlo1, the calculated concentrations of methylglyoxal and GSH were mixed with rTgGlo1. As shown in Fig. 2, the plot for enzymatic activity of rTgGlo1 displayed a curved line and biphasic pattern. Therefore, regression plots were drawn in biphase (Fig. 2; 1/[S] = less than 40 μM and 1/[S] = greater than 40 μM). The K_m_ of phase 1 (K_m1_, 1/[S] = less than 40 μM) and phase 2 (K_m2_, 1/[S] = greater than 40 μM) of rTgGlo1 was 10.0 ± 0.6 and 38.6 ± 1.5 μM, respectively (Table 2). Subsequently, generating seven mutants at glutamate residues were tried by PCR, selected based on the active sites of *P. falciparum* Glo1 [16]. The conserved glutamate residues of Glo1 were previously reported to function in acid–base catalysis during substrate enediolate formation and reprotonation [20, 21]. Five mutants (E92Q, E166Q, R188E, E251Q, and E166Q/E251Q) were successfully generated; however, E324Q and E92Q/E324Q mutants were not prepared despite several trials. The five mutants were evaluated in enzyme activity assays and the enzyme kinetic plots of two mutants also displayed a biphasic pattern as shown in Table 2 and Fig. 2. The K_m_ values of mutants were compared to that of wild-type TgGlo1, and E166Q and E251Q mutants showed higher K_m_ values. Furthermore, K_m_ of the paired mutants (E166Q/E251Q) showed the highest value. In addition, K_cat_ of the paired mutants was 2–10-fold lower than that of wild-type enzyme. These results indicate that the glutamate residues located at positions 166 and 251 are crucial for enzymatic activity. In *P. falciparum* Glo1, the glutamate residue located on 345 amino acid was also reported to be the crucial functional active site. However, we did not succeed in producing the E324Q mutant of TgGlo1. Our results suggest that, the glutamate residues located on 166 and 251 amino acids of TgGlo1 are functional active sites of TgGlo1. The *P. falciparum* Glo1 was reported to possess two functionally paired glutamate residues located at positions 91/345 and 272/161 [16]. As shown in Table 2, the glutamate residues located on 166 and 251 amino acids of TgGlo1 would be functionally coupled. However, it is not clear if glutamate residues on 92 and 345 amino acids are functionally paired, because of the lack of E345Q mutant production. To further evaluate the structural interaction of the residues, studies elucidating the chemical structure of TgGlo1 will be required.

**Table 2:** Enzyme kinetics of recombinant TgGlo1 proteins: a wild type and mutants produced by point-mutagenesis on active sites of TgGlo1

	E92Q			R188E				rTgGlo1	
	Phase 1	Phase 2	E166Q	Phase 1	Phase 2	E251Q	E166Q /E251Q	Phase 1	Phase 2
K_m_ (μM)	11 ± 2.1	42 ± 5.2	173 ± 8.0	14 ± 2.3	42 ± 2.1	94 ± 3.2	190 ± 5.2	10.0 ± 0.6	38.6 ± 1.5
K_cat_ (s^−1^)	159 ± 16	76 ± 9	93 ± 4	654 ± 12	254 ± 20	100 ± 17	114 ± 9	1305 ± 38	257 ± 11

(Phase 1, 1/[S] = less than 40 μM; Phase 2, 1/[S] = more than 40 μM)

### Inhibition of *T. gondii* glyoxalase 1 by curcumin

**Fig. 3:**
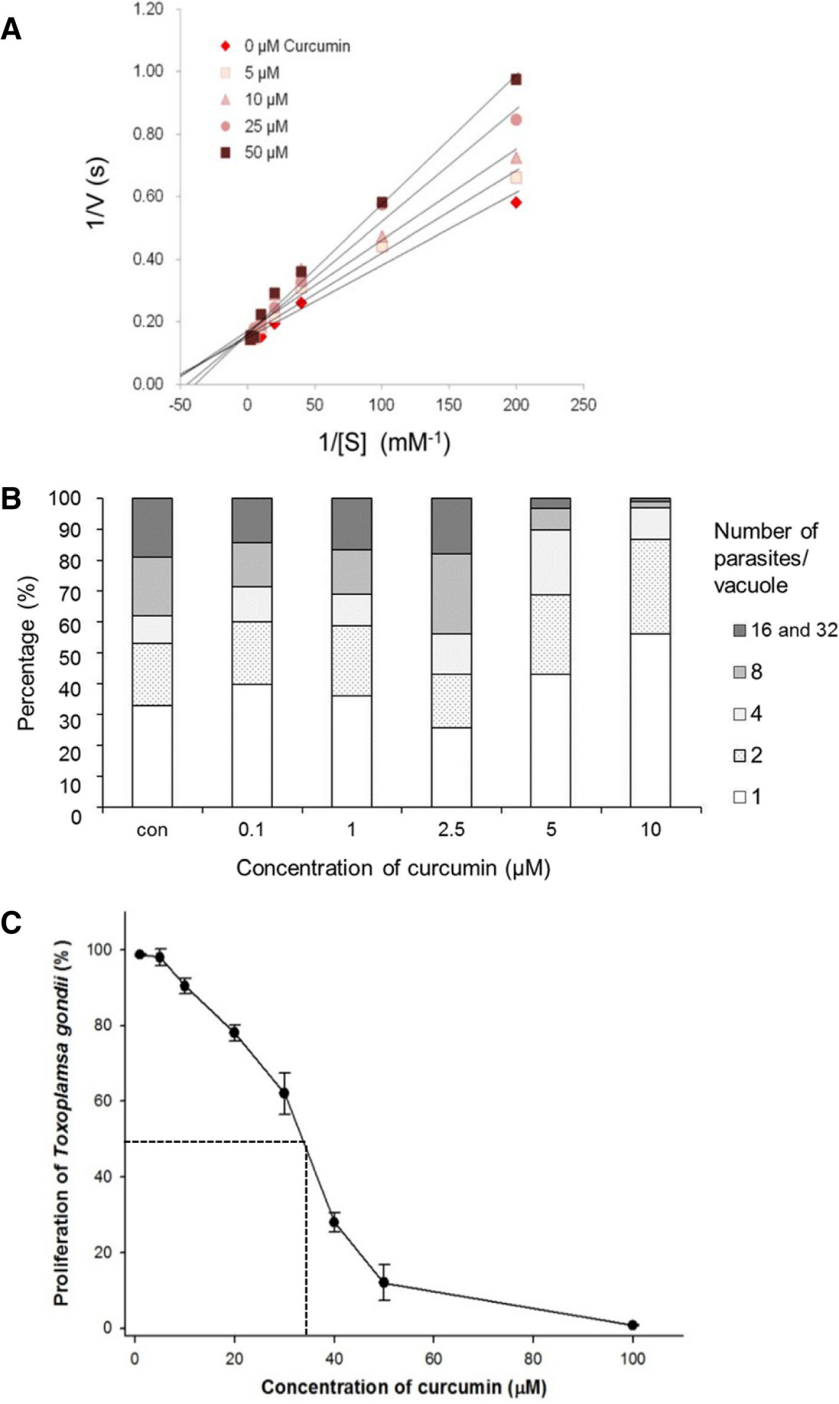
Inhibition of TgGlo1 enzymatic activity and parasite growth of *T. gondii* by curcumin. **a** Inhibitory effects of curcumin on steady-kinetics of rTgGlo1. The purified rTgGlo1 was incubated with decreasing concentrations of curcumin and enzymatic activity was recorded. Enzymatic activity of rTgGlo1 decreased in a dose-dependent manner after treatment with curcumin. **b** Curcumin inhibition study in *T. gondii* cultures. *T. gondii* propagation was inhibited with concentrations of curcumin higher than 5 μM. **c** Curcumin inhibition study in dose–response experiments with *T. gondii*. Proliferation of *T. gondii* was decreased in a dose-dependent manner after treatment with curcumin

Curcumin has been reported to have various pharmacological properties including anti-inflammatory, anti-infectious, and anti-carcinogenic [22]. A number of studies have proposed targets for the pharmacological effects including, transcription factors, enzymes, cytokines, and cell receptors. Glo1 has been considered one of the targets of curcumin in previous studies [12, 17]. Curcumin acts by mimicking hemithioacetal, the enediolate reaction intermediate between methylglyoxal and GSH. Therefore, we investigated the inhibitory effects of curcumin on the enzymatic activity of rTgGlo1 by analyzing the steady-state kinetics at decreasing concentrations of substrate and curcumin. As shown in Fig. 3a, Lineweaver-Burk plots indicate competitive inhibition with a constant maximum reaction velocity. The inhibitory effects were consistent in various timings of curcumin addition, from 1 to 4 min (data not shown). Curcumin has a similar structure to hemithioacetal, and it appears that curcumin directly interacts with the active site of TgGlo1. In addition, the K_i_ value of curcumin against rTgGlo1 was 12.9 ± 0.5 μM, and curcumin inhibition became insignificant at substrate concentrations greater than 125 μM as shown in Additional file 1: Figure S4. Moreover, the enzymatic activity of rTgGlo1 was not completely inhibited by 50 μM curcumin.

We also investigated the effects of curcumin in in vitro cultures of *T. gondii*. The number of vacuoles with more than 16 parasites was remarkably decreased at concentrations higher than 5 μM (Fig. 3b). Specifically, a few vacuoles with many parasites (more than 8) were observed in the cultures treated with 10 μM curcumin, compared to control cultures without curcumin (Fig. 3b). When a broad range of curcumin concentrations (1–100 μM) was tested, the growth of *T. gondii* parasites was inhibited in a dose-dependent manner (Fig. 3c). The IC_50_ of curcumin in *T. gondii* was 38.3 ± 0.9 μM. These results suggest that curcumin may kill the *T. gondii* parasites by interfering with their differentiation. However, it is difficult to conclude that the growth inhibitory effect of curcumin on *T. gondii* was solely the result of targeting TgGlo1, because many effects of curcumin have been reported in the micromolar range [13]. To clarify this point, further evaluation of the direct inhibitory effects of curcumin on the glyoxalase system of *T. gondii* will be needed.

Rapidly propagating cells, including cancer cells and protozoan parasites, consume more glucose and have increased glycolytic flux compared to normal cells, resulting in intracellular levels of methylglyoxal that are toxic to the respective cells [23]. Methylglyoxal has been reported to modify the function of distinct proteins including heat shock protein 27, NF-kB, and glyceraldehyde 3-phosphate dehydrogenase in mammalian cells [24, 25]. Modulation of the proteins causes cell death by depletion of ATP, induction of apoptosis, and increases in levels of reactive oxygen species [26]. In order to remove the toxic methylglyoxal, the cells increase expression of Glo1. Therefore, inhibitors of Glo1 have potential as chemotherapeutic agents.

## Conclusion

In the current study, a cytosolic TgGlo1 was identified and its active sites (E166 and E251) were verified by point mutagenesis. Curcumin inhibited the enzymatic activity of recombinant TgGlo1 and parasite propagation in cultures of *T. gondii*. However, considering the fact that curcumin is known to have many effects on other molecules in the micromolar range, further elucidation of curcumin’s direct inhibition of the glyoxalase system of *T. gondii* will be needed.

## Additional file

Additional file 1: Figure S1. SDS-PAGE of recombinant TgGlo1-His protein (12 % SDS-PAGE gel). Lane M, low molecular mass marker; lane A, recombinant TgGlo1-His protein; lane B, nickel-nitrilotriacetic acid resin after an elution of recombinant TgGlo1-His protein; lane C, *Escherichia coli* expressing recombinant TgGlo1-His after sonication. **Figure S2.** Western blot analysis of recombinant and native TgGlo1. Lane A, recombinant TgGlo1 reacted with anti-TgGlo1 mouse serum; lane B, *Toxoplasma gondii* lysate reacted with anti-TgGlo1 mouse serum; lane C, *T. gondii* lysate reacted with pre-immunized mouse serum. **Figure S3.** Immunofluorescence staining and confocal microscopy for pre-immunized mouse serum. Pre-immunized mouse serum did not react with *Toxoplasma gondii* parasites. Merged image of fluorescent green reactivity and red PI staining of nuclei with phase-contrast images of the parasites showed only red-stained nuclei. **Figure S4.** The inhibitory effects of curcumin on enzymatic activity of rTgGlo1 with different concentrations of hemithioacetal (5–250 μM). (DOC 551 kb)
