# Supplementary material for: Characterization of Toxoplasma gondii glyoxalase 1 and evaluation of inhibitory effects of curcumin on the enzyme and parasite cultures
Source: Parasit Vectors. 2015 Dec 23;8:654. doi: 10.1186/s13071-015-1268-5 (PMC4688987; doi:10.1186/s13071-015-1268-5)
Supplement: Additional file 1: Figure S1. — SDS-PAGE of recombinant TgGlo1-His protein (12 % SDS-PAGE gel). Lane M, low molecular mass marker; lane A, recombinant TgGlo1-His protein; lane B, nickel-nitrilotriacetic acid resin after an elution of recombinant TgGlo1-His protein; lane C, Escherichia coli expressing recombinant TgGlo1-His after sonication. Figure S2. Western blot analysis of recombinant and native TgGlo1. Lane A, recombinant TgGlo1 reacted with anti-TgGlo1 mouse serum; lane B, Toxoplasma gondii lysate reacted with anti-TgGlo1 mouse serum; lane C, T. gondii lysate reacted with pre-immunized mouse serum. Figure S3. Immunofluorescence staining and confocal microscopy for pre-immunized mouse serum. Pre-immunized mouse serum did not react with Toxoplasma gondii parasites. Merged image of fluorescent green reactivity and red PI staining of nuclei with phase-contrast images of the parasites showed only red-stained nuclei. Figure S4. The inhibitory effects of curcumin on enzymatic activity of rTgGlo1 with different concentrations of hemithioacetal (5–250 μM). (DOC 551 kb) [file 13071_2015_1268_MOESM1_ESM.doc]

Supplemental data


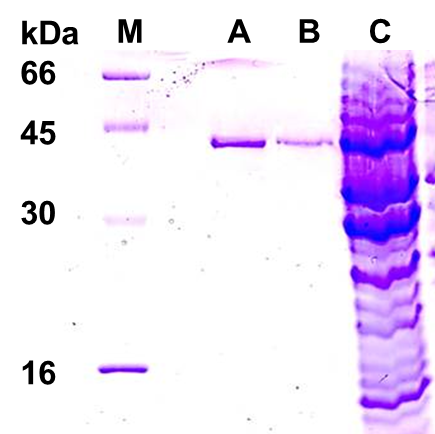


**Figure S1.** SDS-PAGE of recombinant TgGlo1-His protein (12% SDS-PAGE gel).

Lane M, low molecular mass marker; lane A, recombinant TgGlo1-His protein; lane B, nickel-nitrilotriacetic acid resin after an elution of recombinant TgGlo1-His protein; lane C, *Escherichia coli* expressing recombinant TgGlo1-His after sonication.


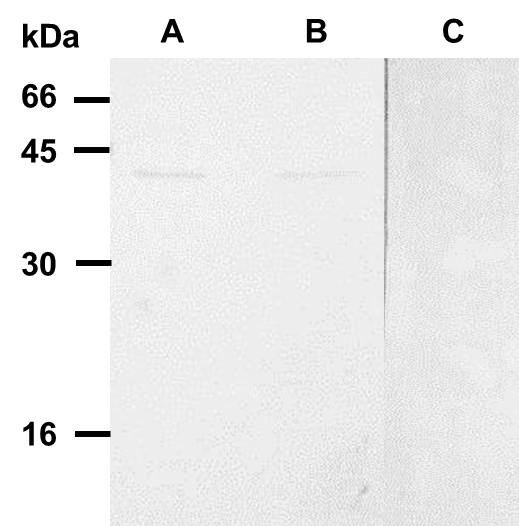


**Figure S2.** Western blot analysis of recombinant and native TgGlo1. Lane A, recombinant TgGlo1 reacted with anti-TgGlo1 mouse serum; lane B, *Toxoplasma gondii* lysate reacted with anti-TgGlo1 mouse serum; lane C, *T*. *gondii* lysate reacted with pre-immunized mouse serum.


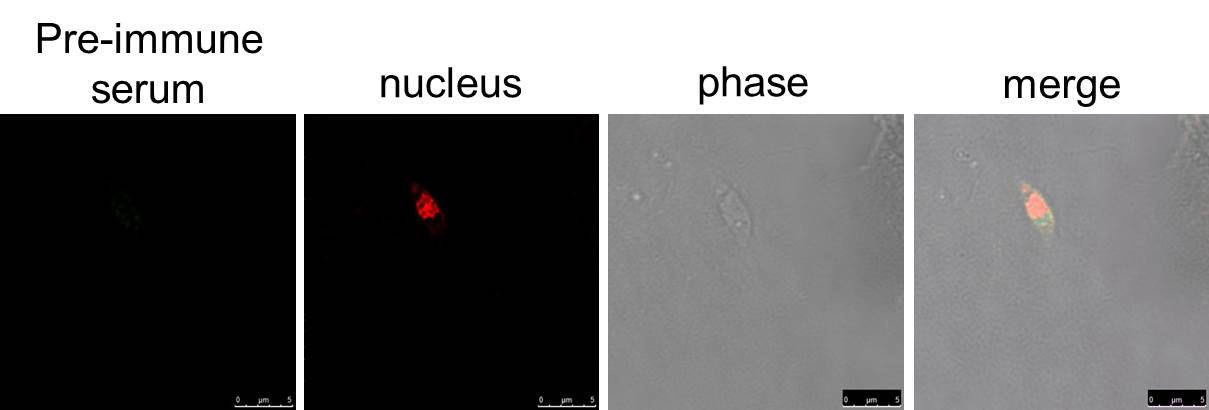


**Figure S3.** Immunofluorescence staining and confocal microscopy for pre-immunized mouse serum. Pre-immunized mouse serum did not react with *Toxoplasma gondii* parasites. Merged image of fluorescent green reactivity and red PI staining of nuclei with phase-contrast images of the parasites showed only red-stained nuclei.

**
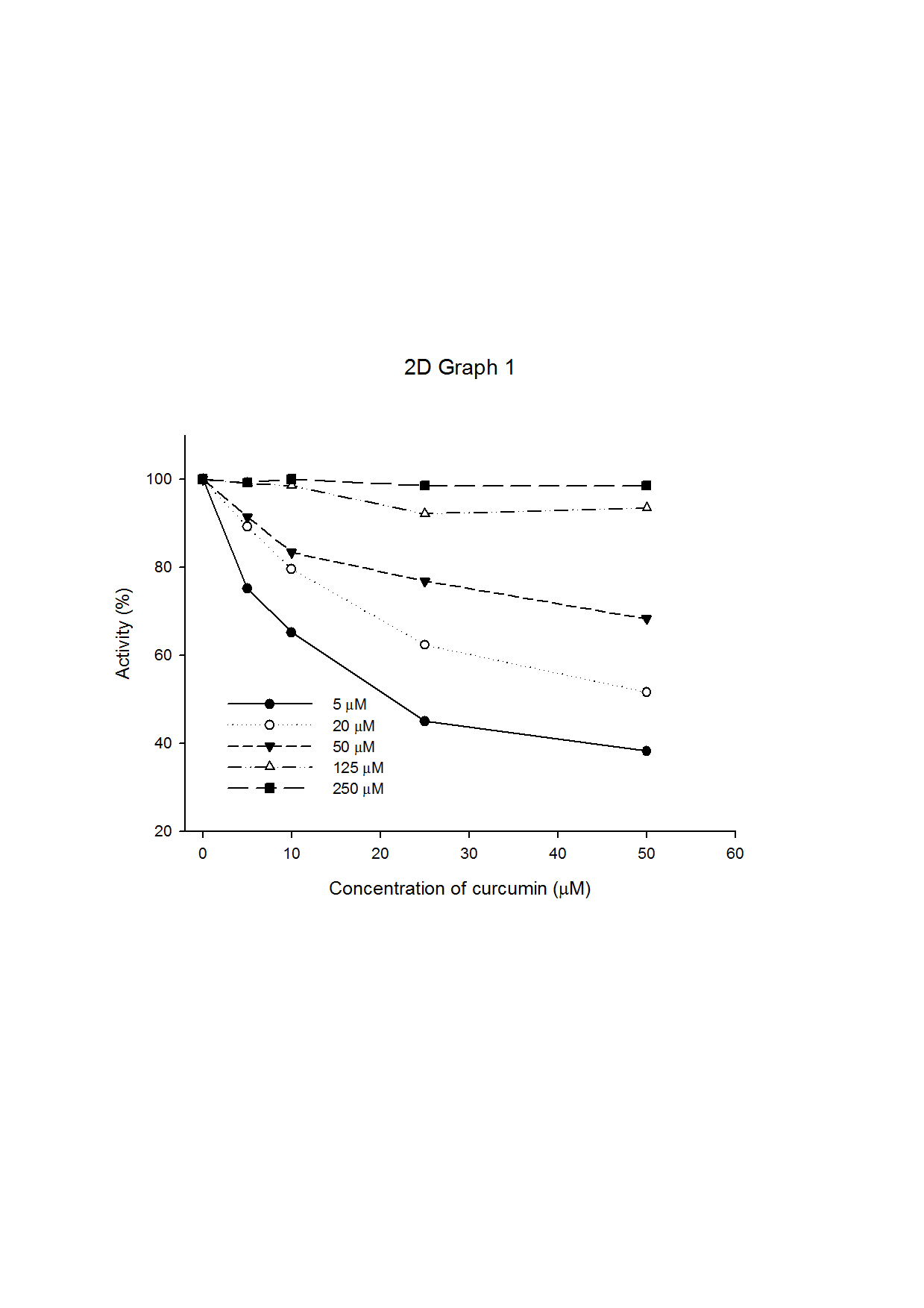
**

**Figure S4.** The inhibitory effects of curcumin on enzymatic activity of rTgGlo1 with different concentrations of hemithioacetal (5–250 μM).
